# Supplementary material for: HIVprotI: an integrated web based platform for prediction and design of HIV proteins inhibitors
Source: J Cheminform. 2018 Mar 9;10:12. doi: 10.1186/s13321-018-0266-y (PMC5845081; doi:10.1186/s13321-018-0266-y)
Supplement: Supplementary file 2 — Additional file 2. Source code of HIVProtI web server. [file 13321_2018_266_MOESM2_ESM.zip › HIVprotI_Source-code/hivprotI/jsme/api_javadoc/deprecated-list.html]

Deprecated List


JavaScript is disabled on your browser.


Skip navigation links


- Package
- Class
- Tree
- Deprecated
- Index
- Help

- Prev
- Next

- Frames
- No Frames

- All Classes

# Deprecated API

## Contents

- Deprecated Methods

- Deprecated Methods

  Method and Description

  export.client.JSME.getNotifyStructuralChangeJSfunction()

  export.client.JSME.setAfterPasteCallback(JavaScriptObject)

  export.client.JSME.setAfterStructureModifiedCallback(JavaScriptObject)

  export.client.JSME.setBeforePasteCallback(JavaScriptObject)

  export.client.JSME.setNotifyAtomHighLightChangeJSfunction(String)

  export.client.JSME.setNotifyStructuralChangeJSfunction(String)

  export.client.JSME.setPrePasteJSfunction(String)

Skip navigation links


- Package
- Class
- Tree
- Deprecated
- Index
- Help

- Prev
- Next

- Frames
- No Frames

- All Classes
